# Diagnosed behavioral health conditions during the perinatal period among a commercially insured population by race/ethnicity, 2008–2020

**DOI:** 10.3389/fpubh.2024.1345442

**Published:** 2024-03-07

**Authors:** Dana C. Beck, Karen Tabb, Anca Tilea, Ashlee J. Vance, Stephanie Hall, Amy Schroeder, Kara Zivin

**Affiliations:** ^1^School of Nursing, University of California, Los Angeles, Los Angeles, CA, United States; ^2^School of Social Work, University of Illinois at Urbana-Champaign, Urbana, IL, United States; ^3^Department of Obstetrics and Gynecology, University of Michigan, Ann Arbor, MI, United States; ^4^Center for Health Policy and Health Services Research, Henry Ford Health, Detroit, MI, United States; ^5^Department of Psychiatry, University of Michigan Medical School, Ann Arbor, MI, United States; ^6^Center for Clinical Management Research, VA Ann Arbor Healthcare System, Ann Arbor, MI, United States

**Keywords:** perinatal mental health, behavioral health, racial disparities, rates of mental health problems, maternal mental health, SUD

## Abstract

**Objective:**

We sought to examine trends in diagnosed behavioral health (BH) conditions [mental health (MH) disorders or substance use disorders (SUD)] among pregnant and postpartum individuals between 2008–2020. We then explored the relationship between BH conditions and race/ethnicity, acknowledging race/ethnicity as a social construct that influences health disparities.

**Methods:**

This study included delivering individuals, aged 15–44 years, and continuously enrolled in a single commercial health insurance plan for 1 year before and 1 year following delivery between 2008–2020. We used BH conditions as our outcome based on relevant ICD 9/10 codes documented during pregnancy or the postpartum year.

**Results:**

In adjusted analyses, white individuals experienced the highest rates of BH conditions, followed by Black, Hispanic, and Asian individuals, respectively. Asian individuals had the largest increase in BH rates, increasing 292%. White individuals had the smallest increase of 192%. The trend remained unchanged even after adjusting for age and Bateman comorbidity score, the trend remained unchanged.

**Conclusions:**

The prevalence of diagnosed BH conditions among individuals in the perinatal and postpartum periods increased over time. As national efforts continue to work toward improving perinatal BH, solutions must incorporate the needs of diverse populations to avert preventable morbidity and mortality.

## 1 Introduction

Behavioral health (BH) disorders are a leading contributor to perinatal mortality in the U.S. (1). Behavioral health problems encompass a broad umbrella of conditions, such as mental health (MH) disorders, substance use disorders (SUD), and co-occurring MH and SUD (2). Ongoing, national efforts aim to improve clinical practice and health policy to support perinatal individuals with BH conditions (2). Equity-focused practice and policy level interventions require a data-driven understanding of trends in BH conditions by race/ethnicity. This knowledge may help foster solutions related to inequitable access to and use of treatment due to systemic racism (3). Few studies have used a racially and ethnically diverse national sample to describe diagnoses of BH conditions during the perinatal period (pregnancy and the postpartum year). Therefore, this study characterized BH (MH or SUD) diagnosis trends during the perinatal period among a racially and ethnically diverse, commercially insured population between 2008–2020.

## 2 Method

We examined trends in BH diagnoses identified during the perinatal period (pregnancy and 1 year postpartum) among individuals aged 15–44 using Optum Clinformatics Data Mart (CDM). CDM is a de-identified administrative medical claims database derived from a large claims data warehouse for members across 50 states. We identified individuals with deliveries from 2008 to 2020 and restricted the sample to those with continuous enrollment in a single employer-based health plan for at least 1 year before and 1 year after delivery. We identified delivery hospitalizations using standardized International Classification of Disease-9th and 10th Revision-Clinical Modification (ICD-9-CM and ICD-10-CM) diagnosis and procedure codes.

We defined BH diagnosis as evidence of any MH or SUD ICD-9/10 diagnosis code present in at least one inpatient claim or two outpatient claims during the year prior to the delivery or during the year after the delivery. We used Healthcare Cost and Utilization Project (HCUP) codes definitions to identify diagnoses (4).

We summarized demographic characteristics for all individuals with evidence of a BH disorder including any MH, any SUD, or both MH and SUD. We applied logistic regression to calculate the probability of BH diagnosis, adjusting for race, age, geographic region, insurance type, income, delivery mode, and clinical comorbidity as measured using the Bateman Comorbidity Index (5). We calculated the predicted probabilities of each outcome rate for each year of the study period by race/ethnicity to describe trends in BH diagnoses per race/ethnicity group. We used two-sided statistical tests with an alpha level of 0.05 for all statistical analyses. We performed all claims data management in SAS version 9.4 (SAS Institute) and statistical analyses in R (R Core Team).

This study was approved by the study site's Institutional Review Board (HUM00164685).

## 3 Results

We identified 736,325 deliveries from 621,148 commercially insured delivering individuals between 2008 and 2020. Of these, 202,489 (27.6%) had evidence of a BH diagnosis in either the prenatal or the postpartum year, 26.1% had evidence of any MH diagnosis, and nearly 4.0% had evidence of any SUD diagnosis (Table 1).

**Table 1:** Descriptive characteristics of the study cohort, for years 2008 and 2020, by different BH group (any MH, ANY SUD, MH or SUD, MH, and SUD).

**Characteristic**	**2008**				**2020**			
	**Any MH**	**Any SUD**	**MH or SUD**	**MH and SUD**	**Any MH**	**Any SUD**	**MH or SUD**	**MH and SUD**
	***N** =* **12,524**	***N** =* **1,205**	***N** =* **13,007**	***N** =* **721**	***N** =* **19,879**	***N** =* **3,287**	***N** =* **20,985**	***N** =* **2,181**
**Age group**								
15–18	263 (23.91%)	66 (6.00%)	278 (25.27%)	51 (4.64%)	131 (53.04%)	46 (18.62%)	142 (57.49%)	35 (14.17%)
19–26	1,985 (20.00%)	299 (3.01%)	2,085 (21.01%)	199 (2.01%)	3,017 (41.92%)	966 (13.42%)	3,341 (46.42%)	642 (8.92%)
27–34	6,428 (18.75%)	530 (1.55%)	6,668 (19.45%)	290 (0.85%)	10,343 (37.63%)	1,395 (5.07%)	10,851 (39.48%)	887 (3.23%)
35–39	2,923 (20.15%)	240 (1.65%)	3,029 (20.88%)	133 (0.92%)	5,139 (39.08%)	672 (5.11%)	5,345 (40.65%)	466 (3.54%)
40+	925 (23.70%)	70 (1.79%)	947 (24.26%)	48 (1.23%)	1,249 (39.80%)	208 (6.63%)	1,306 (41.62%)	151 (4.81%)
**Race**								
Unknown/missing	1,316 (18.91%)	33 (0.78%)	369 (8.73%)	12 (0.28%)	1,823 (33.12%)	75 (2.21%)	864 (25.44%)	47 (1.38%)
Asian	348 (8.23%)	124 (2.18%)	1,005 (17.64%)	68 (1.19%)	836 (24.62%)	483 (11.42%)	1,850 (43.72%)	288 (6.81%)
Black	949 (16.66%)	122 (1.53%)	1,353 (17.00%)	61 (0.77%)	1,655 (39.12%)	362 (5.58%)	2,399 (36.95%)	206 (3.17%)
Hispanic	1,292 (16.24%)	132 (1.90%)	1,372 (19.71%)	76 (1.09%)	2,243 (34.54%)	283 (5.14%)	1,936 (35.17%)	170 (3.09%)
White	8,619 (22.18%)	794 (2.04%)	8,908 (22.92%)	504 (1.30%)	13,322 (42.16%)	2,084 (6.60%)	13,936 (44.11%)	1,470 (4.65%)
**Division**								
Great lakes/northern plains	3,235 (21.38%)	278 (1.84%)	3,331 (22.02%)	182 (1.20%)	5,773 (40.43%)	919 (6.44%)	6,086 (42.62%)	606 (4.24%)
Mountain	1,200 (20.84%)	119 (2.07%)	1,251 (21.73%)	68 (1.18%)	2,120 (39.35%)	353 (6.55%)	2,242 (41.62%)	231 (4.29%)
Northeast	1,340 (20.75%)	112 (1.73%)	1,392 (21.55%)	60 (0.93%)	2,063 (40.48%)	306 (6.00%)	2,171 (42.60%)	198 (3.89%)
Pacific	965 (13.52%)	89 (1.25%)	1,006 (14.10%)	48 (0.67%)	1,851 (34.59%)	160 (2.99%)	1,914 (35.76%)	97 (1.81%)
Southeast	5,779 (19.81%)	607 (2.08%)	6,022 (20.64%)	363 (1.24%)	8,054 (38.39%)	1,546 (7.37%)	8,554 (40.77%)	1,046 (4.99%)
Unknown/Missing	^*^	^*^	^*^	^*^	^*^	^*^	^*^	^*^
**Insurance**								
EPO	2,152 (19.48%)	189 (1.71%)	2,229 (20.17%)	112 (1.01%)	2,443 (38.15%)	450 (7.03%)	2,618 (40.88%)	275 (4.29%)
HMO	1,558 (18.03%)	155 (1.79%)	1,608 (18.61%)	104 (1.20%)	2,073 (37.99%)	359 (6.58%)	2,184 (40.03%)	248 (4.55%)
IND	^*^	^*^	^*^	^*^	^*^	^*^	^*^	^*^
OTH	49 (32.03%)	10 (6.54%)	51 (33.33%)	8 (5.23%)	475 (44.06%)	89 (8.26%)	495 (45.92%)	69 (6.40%)
POS	8,295 (19.84%)	805 (1.93%)	8,635 (20.65%)	465 (1.11%)	14,646 (38.91%)	2,348 (6.24%)	15,434 (41.01%)	1,560 (4.14%)
PPO	465 (22.77%)	45 (2.20%)	478 (23.41%)	32 (1.57%)	242 (37.69%)	41 (6.39%)	254 (39.56%)	29 (4.52%)
**Federal poverty level**								
Unknown/Missing	2,848 (19.56%)	310 (2.13%)	2,963 (20.35%)	195 (1.34%)	2,504 (32.38%)	423 (5.47%)	2,669 (34.51%)	258 (3.34%)
< 250% FPL	1,476 (20.37%)	181 (2.50%)	1,540 (21.25%)	117 (1.61%)	4,877 (40.30%)	1,238 (10.23%)	5,328 (44.03%)	787 (6.50%)
250–400% FPL	2,007 (20.45%)	204 (2.08%)	2,092 (21.32%)	118 (1.20%)	5,213 (39.44%)	856 (6.48%)	5,479 (41.45%)	590 (4.46%)
Above 400% FPL	6,193 (19.30%)	510 (1.59%)	6,412 (19.98%)	291 (0.91%)	7,285 (40.10%)	770 (4.24%)	7,509 (41.33%)	546 (3.01%)
**Delivery mode**								
Vaginal	7,494 (18.61%)	684 (1.70%)	7,753 (19.25%)	425 (1.06%)	12,800 (37.43%)	2,001 (5.85%)	13,499 (39.48%)	1,302 (3.81%)
C-section	5,030 (21.47%)	521 (2.22%)	5,254 (22.42%)	296 (1.26%)	7,079 (41.58%)	1,286 (7.55%)	7,486 (43.97%)	879 (5.16%)
**Bateman score**								
Bateman (0,1)	11,401 (19.12%)	1,014 (1.70%)	11,818 (19.82%)	596 (1.00%)	15,132 (37.12%)	1,992 (4.89%)	15,859 (38.91%)	1,265 (3.10%)
Bateman 2+	1,123 (27.43%)	191 (4.67%)	1,189 (29.04%)	125 (3.05%)	4,747 (45.40%)	1,295 (12.38%)	5,126 (49.02%)	916 (8.76%)

^*^Cells of *N* < 11 redacted to preserve confidentiality.

**Figure 1:**
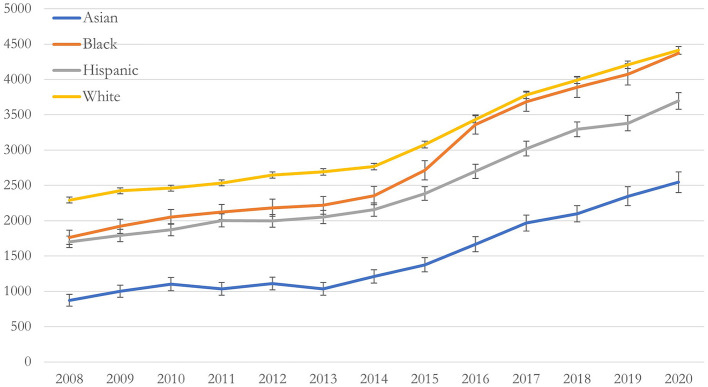
Unadjusted predicted rate of behavioral health conditions per 10,000 deliveries, by race/ethnicity, between 2008–2020 in a national commercially insured sample of perinatal individuals. Unadjusted rate of behavioral health (BH) conditions by race/ethnicity.

**Figure 2:**
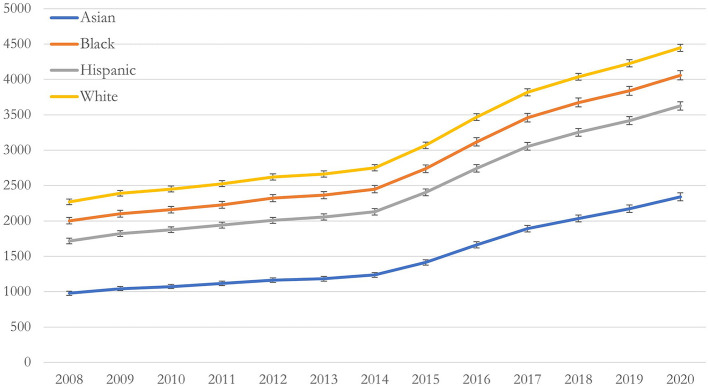
Adjusted predicted rate of behavioral health conditions per 10,000 deliveries, by race/ethnicity, between 2008–2020 in a national commercially insured sample of perinatal individuals. Adjusted rates of BH conditions by race/ethnicity adjusting for age, geographic region, insurance type, income, delivery mode, and Bateman index score.

Figure 1 shows the unadjusted and adjusted BH rates per 10,000 deliveries grouped by race/ethnicity. All groups increased at the same rate over the study period. White individuals had the highest rates of BH diagnoses, followed by Black, Hispanic, and Asian groups, respectively. Asian individuals had the largest increase in BH rates from 2008 to 2020 with a 292% increase from 872.7 (95% CI: 787–957) per 10,000 individuals in 2008 to 2,544 (95% CI: 2,397–2,690) per 10,000 individuals in 2020 (Figure 1), while white individuals had the smallest increase in BH rates, with a 192% increase from 2,291 (95% CI: 2,250–2,333) per 10,000 in 2008 to 4,410 (95% CI: 4,355–4,465) per 10,000 in 2020 (Figure 1). After adjusting for age and comorbidity, the trend remained unchanged (Figure 2).

## 4 Discussion

This study found increasing rates of perinatal BH diagnoses during pregnancy and the postpartum year with the greatest increases among Asian individuals. As national efforts to address BH related perinatal morbidity and mortality continue, the needs of diverse groups must be addressed in the context of the systemic drivers of health disparities, including systemic racism (2). This study highlights the need for clinicians to be attentive to the BH symptoms and needs of minoritized groups during pregnancy and the postpartum year, particularly for Asian individuals. A limitation of this study is the use of a commercially insured population, and trends in diagnoses might be different among publicly insured (e.g., Medicaid insured) individuals.

This study supports the need for equitable screening, referral, and treatment of perinatal BH conditions during pregnancy and the postpartum year. Efforts continue toward implementation of structural-level supports to address the health impacts of systemic racism among perinatal individuals, including Medicaid expansion through the full postpartum year and paid parental leave policies (2). While recognizing and diagnosing BH conditions is essential so individuals can receive treatment and ongoing support, individual/health systems interventions must be coupled with structural-level policy change to counteract the health effects of systemic racism.

## Data availability statement

The data analyzed in this study is subject to the following licenses/restrictions: the data that support the findings of this study are available from Optum's de-identified Clinformatics^®^ Data Mart Database (CDM). Restrictions apply to the availability of these data, which were used under license for this study, and thus are not publicly available. Requests to access these datasets should be directed to Optum Life Sciences, https://www.optum.com/business/life-sciences/real-world-data/claims-data.html.

## Ethics statement

The studies involving humans were approved by University of Michigan Institutional Review Board. The studies were conducted in accordance with the local legislation and institutional requirements. Written informed consent for participation was not required from the participants or the participants' legal guardians/next of kin in accordance with the national legislation and institutional requirements.

## Author contributions

DB: Writing – original draft. KT: Writing – original draft, Writing – review & editing. AT: Writing – original draft, Writing – review & editing. AV: Writing – review & editing. SH: Writing – review & editing. AS: Writing – review & editing. KZ: Writing – review & editing.
